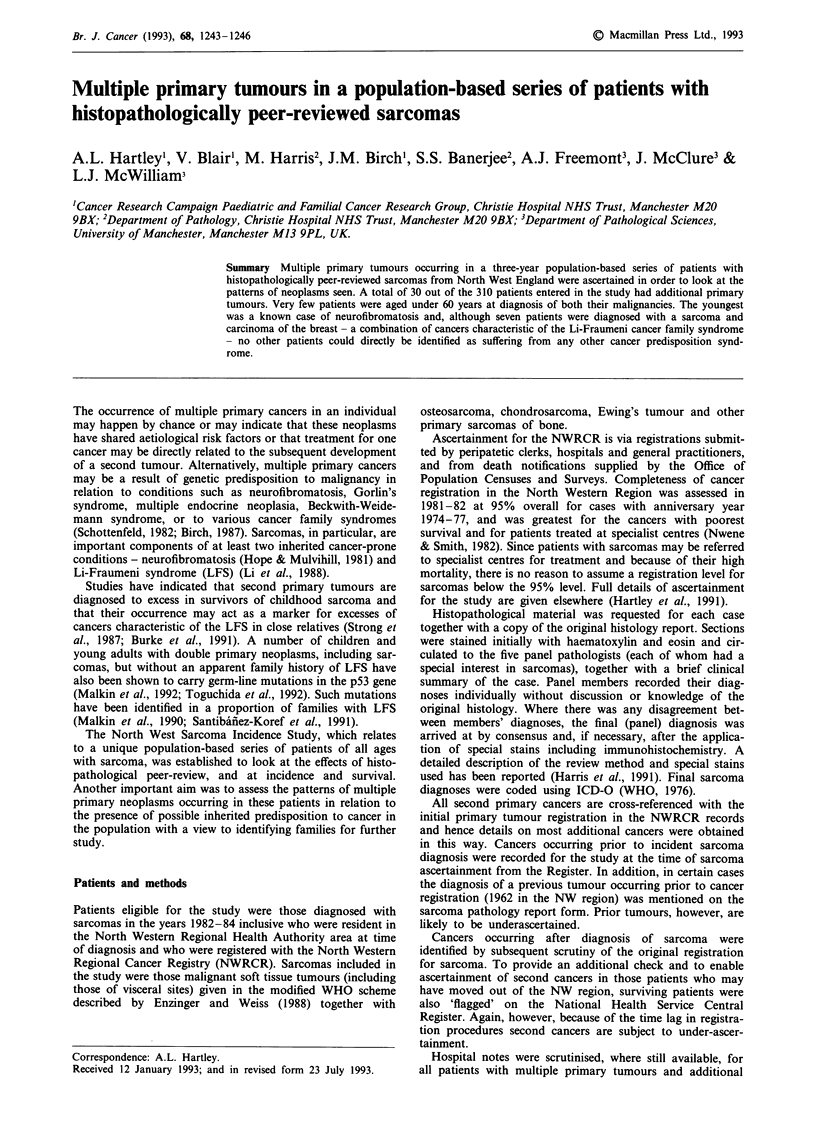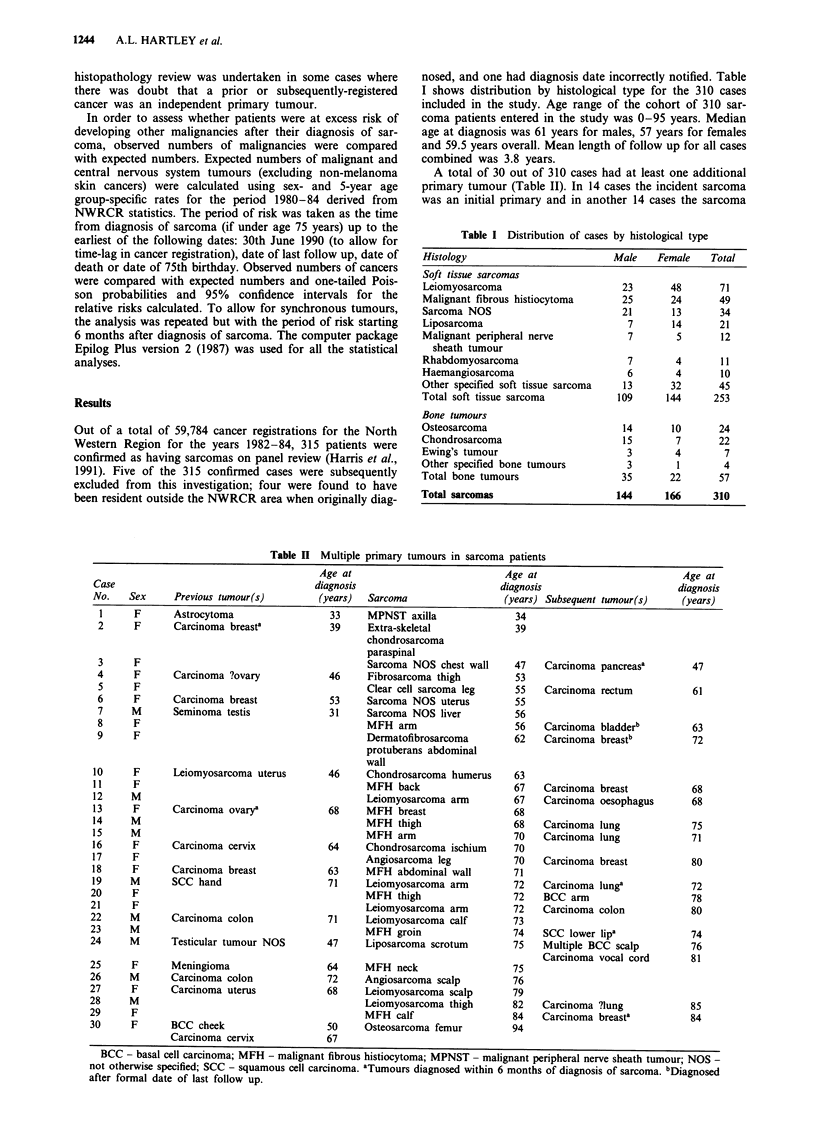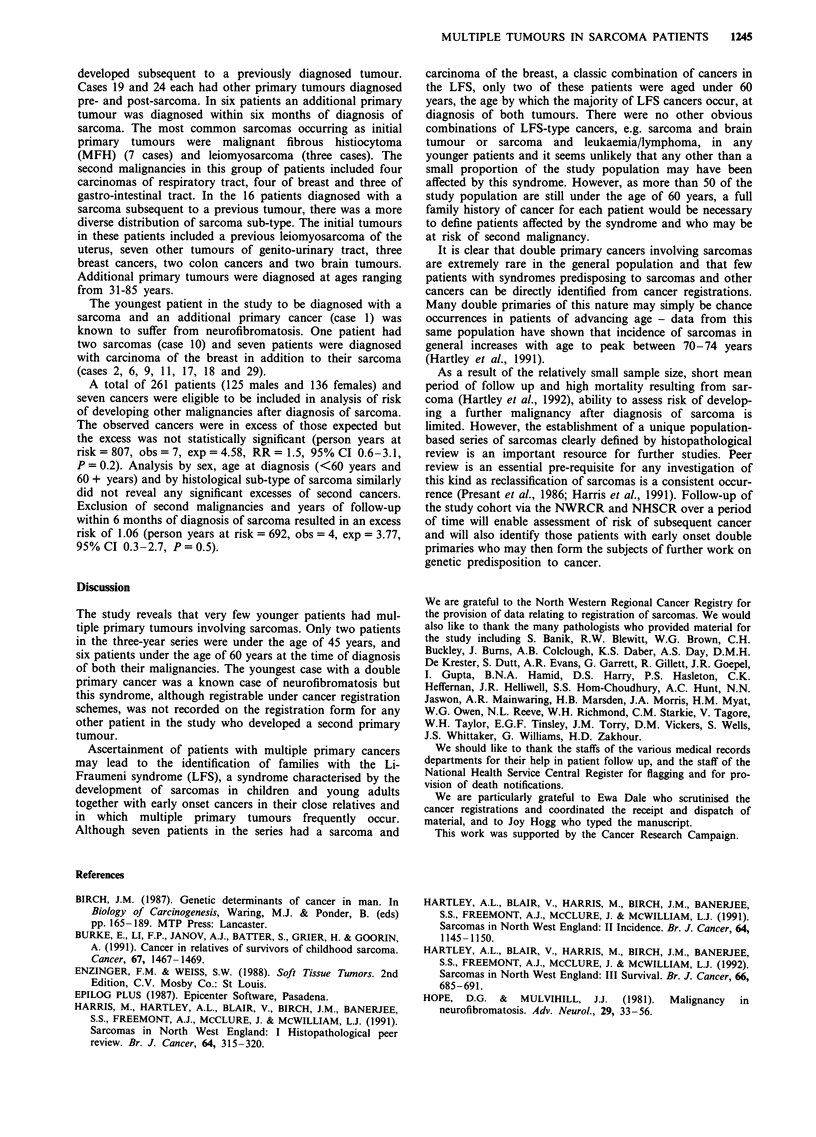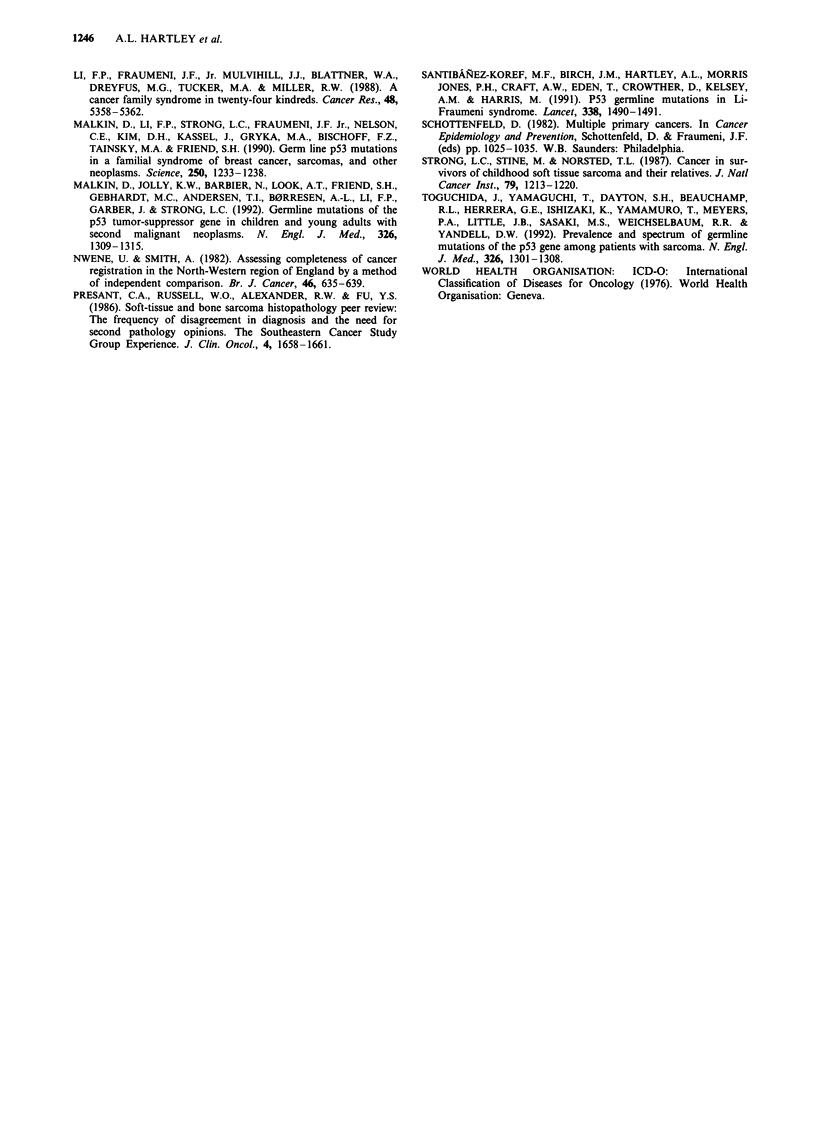# Multiple primary tumours in a population-based series of patients with histopathologically peer-reviewed sarcomas.

**DOI:** 10.1038/bjc.1993.512

**Published:** 1993-12

**Authors:** A. L. Hartley, V. Blair, M. Harris, J. M. Birch, S. S. Banerjee, A. J. Freemont, J. McClure, L. J. McWilliam

**Affiliations:** Cancer Research Campaign Paediatric and Familial Cancer Research Group, Christie Hospital NHS Trust, Manchester, UK.

## Abstract

Multiple primary tumours occurring in a three-year population-based series of patients with histopathologically peer-reviewed sarcomas from North West England were ascertained in order to look at the patterns of neoplasms seen. A total of 30 out of the 310 patients entered in the study had additional primary tumours. Very few patients were aged under 60 years at diagnosis of both their malignancies. The youngest was a known case of neurofibromatosis and, although seven patients were diagnosed with a sarcoma and carcinoma of the breast--a combination of cancers characteristic of the Li-Fraumeni cancer family syndrome--no other patients could directly be identified as suffering from any other cancer predisposition syndrome.


 © Macmillan Press Ltd., 1993

### Multiple primary tumours in a population-based series of patients with histopathologically peer-reviewed sarcomas

A.L. Hartley[1], V. Blair[1], M. Harris[2], J.M. Birch[1], S.S. Banerjee[2], A.J. Freemont[3], J. McClure[3] & L.J. McWilliam[3]

[1]*Cancer Research Campaign Paediatric and Familial Cancer Research Group, Christie Hospital NHS Trust, Manchester M20 9BX;* [2]*Department of Pathology, Christie Hospital NHS Trust, Manchester M20 9BX;* [3]*Department of Pathological Sciences, University of Manchester, Manchester M13 9PL, UK.*

**Summary** Multiple primary tumours occurring in a three-year population-based series of patients with histopathologically peer-reviewed sarcomas from North West England were ascertained in order to look at the patterns of neoplasms seen. A total of 30 out of the 310 patients entered in the study had additional primary tumours. Very few patients were aged under 60 years at diagnosis of both their malignancies. The youngest was a known case of neurofibromatosis and, although seven patients were diagnosed with a sarcoma and carcinoma of the breast – a combination of cancers characteristic of the Li-Fraumeni cancer family syndrome – no other patients could directly be identified as suffering from any other cancer predisposition syndrome.

The occurrence of multiple primary cancers in an individual may happen by chance or may indicate that these neoplasms have shared aetiological risk factors or that treatment for one cancer may be directly related to the subsequent development of a second tumour. Alternatively, multiple primary cancers may be a result of genetic predisposition to malignancy in relation to conditions such as neurofibromatosis, Gorlin's syndrome, multiple endocrine neoplasia, Beckwith-Weidemann syndrome, or to various cancer family syndromes (Schottenfeld, 1982; Birch, 1987). Sarcomas, in particular, are important components of at least two inherited cancer-prone conditions – neurofibromatosis (Hope & Mulvihill, 1981) and Li-Fraumeni syndrome (LFS) (Li *et al.*, 1988).

Studies have indicated that second primary tumours are diagnosed to excess in survivors of childhood sarcoma and that their occurrence may act as a marker for excesses of cancers characteristic of the LFS in close relatives (Strong *et al.*, 1987; Burke *et al.*, 1991). A number of children and young adults with double primary neoplasms, including sarcomas, but without an apparent family history of LFS have also been shown to carry germ-line mutations in the p53 gene (Malkin *et al.*, 1992; Toguchida *et al.*, 1992). Such mutations have been identified in a proportion of families with LFS (Malkin *et al.*, 1990; Santibáñez-Koref *et al.*, 1991).

The North West Sarcoma Incidence Study, which relates to a unique population-based series of patients of all ages with sarcoma, was established to look at the effects of histopathological peer-review, and at incidence and survival. Another important aim was to assess the patterns of multiple primary neoplasms occurring in these patients in relation to the presence of possible inherited predisposition to cancer in the population with a view to identifying families for further study.

### Patients and methods

Patients eligible for the study were those diagnosed with sarcomas in the years 1982–84 inclusive who were resident in the North Western Regional Health Authority area at time of diagnosis and who were registered with the North Western Regional Cancer Registry (NWRCR). Sarcomas included in the study were those malignant soft tissue tumours (including those of visceral sites) given in the modified WHO scheme described by Enzinger and Weiss (1988) together with osteosarcoma, chondrosarcoma, Ewing's tumour and other primary sarcomas of bone.

Ascertainment for the NWRCR is via registrations submitted by peripatetic clerks, hospitals and general practitioners, and from death notifications supplied by the Office of Population Censuses and Surveys. Completeness of cancer registration in the North Western Region was assessed in 1981–82 at 95% overall for cases with anniversary year 1974–77, and was greatest for the cancers with poorest survival and for patients treated at specialist centres (Nwene & Smith, 1982). Since patients with sarcomas may be referred to specialist centres for treatment and because of their high mortality, there is no reason to assume a registration level for sarcomas below the 95% level. Full details of ascertainment for the study are given elsewhere (Hartley *et al.*, 1991).

Histopathological material was requested for each case together with a copy of the original histology report. Sections were stained initially with haematoxylin and eosin and circulated to the five panel pathologists (each of whom had a special interest in sarcomas), together with a brief clinical summary of the case. Panel members recorded their diagnoses individually without discussion or knowledge of the original histology. Where there was any disagreement between members' diagnoses, the final (panel) diagnosis was arrived at by consensus and, if necessary, after the application of special stains including immunohistochemistry. A detailed description of the review method and special stains used has been reported (Harris *et al.*, 1991). Final sarcoma diagnoses were coded using ICD-O (WHO, 1976).

All second primary cancers are cross-referenced with the initial primary tumour registration in the NWRCR records and hence details on most additional cancers were obtained in this way. Cancers occurring prior to incident sarcoma diagnosis were recorded for the study at the time of sarcoma ascertainment from the Register. In addition, in certain cases the diagnosis of a previous tumour occurring prior to cancer registration (1962 in the NW region) was mentioned on the sarcoma pathology report form. Prior tumours, however, are likely to be underascertained.

Cancers occurring after diagnosis of sarcoma were identified by subsequent scrutiny of the original registration for sarcoma. To provide an additional check and to enable ascertainment of second cancers in those patients who may have moved out of the NW region, surviving patients were also 'flagged' on the National Health Service Central Register. Again, however, because of the time lag in registration procedures second cancers are subject to under-ascertainment.

Hospital notes were scrutinised, where still available, for all patients with multiple primary tumours and additional

Correspondence: A.L. Hartley.
Received 12 January 1993; and in revised form 23 July 1993.

histopathology review was undertaken in some cases where there was doubt that a prior or subsequently-registered cancer was an independent primary tumour.

In order to assess whether patients were at excess risk of developing other malignancies after their diagnosis of sarcoma, observed numbers of malignancies were compared with expected numbers. Expected numbers of malignant and central nervous system tumours (excluding non-melanoma skin cancers) were calculated using sex- and 5-year age group-specific rates for the period 1980–84 derived from NWRCR statistics. The period of risk was taken as the time from diagnosis of sarcoma (if under age 75 years) up to the earliest of the following dates: 30th June 1990 (to allow for time-lag in cancer registration), date of last follow up, date of death or date of 75th birthday. Observed numbers of cancers were compared with expected numbers and one-tailed Poisson probabilities and 95% confidence intervals for the relative risks calculated. To allow for synchronous tumours, the analysis was repeated but with the period of risk starting 6 months after diagnosis of sarcoma. The computer package Epilog Plus version 2 (1987) was used for all the statistical analyses.

### Results

Out of a total of 59,784 cancer registrations for the North Western Region for the years 1982–84, 315 patients were confirmed as having sarcomas on panel review (Harris *et al.*, 1991). Five of the 315 confirmed cases were subsequently excluded from this investigation; four were found to have been resident outside the NWRCR area when originally diagnosed, and one had diagnosis date incorrectly notified. Table I shows distribution by histological type for the 310 cases included in the study. Age range of the cohort of 310 sarcoma patients entered in the study was 0–95 years. Median age at diagnosis was 61 years for males, 57 years for females and 59.5 years overall. Mean length of follow up for all cases combined was 3.8 years.

A total of 30 out of 310 cases had at least one additional primary tumour (Table II). In 14 cases the incident sarcoma was an initial primary and in another 14 cases the sarcoma

**Table I** Distribution of cases by histological type

| *Histology* | *Male* | *Female* | *Total* |
|---|---|---|---|
| *Soft tissue sarcomas* | | | |
| Leiomyosarcoma | 23 | 48 | 71 |
| Malignant fibrous histiocytoma | 25 | 24 | 49 |
| Sarcoma NOS | 21 | 13 | 34 |
| Liposarcoma | 7 | 14 | 21 |
| Malignant peripheral nerve sheath tumour | 7 | 5 | 12 |
| Rhabdomyosarcoma | 7 | 4 | 11 |
| Haemangiosarcoma | 6 | 4 | 10 |
| Other specified soft tissue sarcoma | 13 | 32 | 45 |
| Total soft tissue sarcoma | 109 | 144 | 253 |
| *Bone tumours* | | | |
| Osteosarcoma | 14 | 10 | 24 |
| Chondrosarcoma | 15 | 7 | 22 |
| Ewing's tumour | 3 | 4 | 7 |
| Other specified bone tumours | 3 | 1 | 4 |
| Total bone tumours | 35 | 22 | 57 |
| **Total sarcomas** | **144** | **166** | **310** |

**Table II** Multiple primary tumours in sarcoma patients

| *Case No.* | *Sex* | *Previous tumour(s)* | *Age at diagnosis (years)* | *Sarcoma* | *Age at diagnosis (years)* | *Subsequent tumour(s)* | *Age at diagnosis (years)* |
|---|---|---|---|---|---|---|---|
| 1 | F | Astrocytoma | 33 | MPNST axilla | 34 | | |
| 2 | F | Carcinoma breast[a] | 39 | Extra-skeletal chondrosarcoma paraspinal | 39 | | |
| 3 | F | | | Sarcoma NOS chest wall | 47 | Carcinoma pancreas[a] | 47 |
| 4 | F | Carcinoma ?ovary | 46 | Fibrosarcoma thigh | 53 | | |
| 5 | F | | | Clear cell sarcoma leg | 55 | Carcinoma rectum | 61 |
| 6 | F | Carcinoma breast | 53 | Sarcoma NOS uterus | 55 | | |
| 7 | M | Seminoma testis | 31 | Sarcoma NOS liver | 56 | | |
| 8 | F | | | MFH arm | 56 | Carcinoma bladder[b] | 63 |
| 9 | F | | | Dermatofibrosarcoma protuberans abdominal wall | 62 | Carcinoma breast[b] | 72 |
| 10 | F | Leiomyosarcoma uterus | 46 | Chondrosarcoma humerus | 63 | | |
| 11 | F | | | MFH back | 67 | Carcinoma breast | 68 |
| 12 | M | | | Leiomyosarcoma arm | 67 | Carcinoma oesophagus | 68 |
| 13 | F | Carcinoma ovary[a] | 68 | MFH breast | 68 | | |
| 14 | M | | | MFH thigh | 68 | Carcinoma lung | 75 |
| 15 | M | | | MFH arm | 70 | Carcinoma lung | 71 |
| 16 | F | Carcinoma cervix | 64 | Chondrosarcoma ischium | 70 | | |
| 17 | F | | | Angiosarcoma leg | 70 | Carcinoma breast | 80 |
| 18 | F | Carcinoma breast | 63 | MFH abdominal wall | 71 | | |
| 19 | M | SCC hand | 71 | Leiomyosarcoma arm | 72 | Carcinoma lung[a] | 72 |
| 20 | F | | | MFH thigh | 72 | BCC arm | 78 |
| 21 | F | | | Leiomyosarcoma arm | 72 | Carcinoma colon | 80 |
| 22 | M | Carcinoma colon | 71 | Leiomyosarcoma calf | 73 | | |
| 23 | M | | | MFH groin | 74 | SCC lower lip[a] | 74 |
| 24 | M | Testicular tumour NOS | 47 | Liposarcoma scrotum | 75 | Multiple BCC scalp<br>Carcinoma vocal cord | 76<br>81 |
| 25 | F | Meningioma | 64 | MFH neck | 75 | | |
| 26 | M | Carcinoma colon | 72 | Angiosarcoma scalp | 76 | | |
| 27 | F | Carcinoma uterus | 68 | Leiomyosarcoma scalp | 79 | | |
| 28 | M | | | Leiomyosarcoma thigh | 82 | Carcinoma ?lung | 85 |
| 29 | F | | | MFH calf | 84 | Carcinoma breast[a] | 84 |
| 30 | F | BCC cheek<br>Carcinoma cervix | 50<br>67 | Osteosarcoma femur | 94 | | |

BCC – basal cell carcinoma; MFH – malignant fibrous histiocytoma; MPNST – malignant peripheral nerve sheath tumour; NOS – not otherwise specified; SCC – squamous cell carcinoma. [a]Tumours diagnosed within 6 months of diagnosis of sarcoma. [b]Diagnosed after formal date of last follow up.

developed subsequent to a previously diagnosed tumour. Cases 19 and 24 each had other primary tumours diagnosed pre- and post-sarcoma. In six patients an additional primary tumour was diagnosed within six months of diagnosis of sarcoma. The most common sarcomas occurring as initial primary tumours were malignant fibrous histiocytoma (MFH) (7 cases) and leiomyosarcoma (three cases). The second malignancies in this group of patients included four carcinomas of respiratory tract, four of breast and three of gastro-intestinal tract. In the 16 patients diagnosed with a sarcoma subsequent to a previous tumour, there was a more diverse distribution of sarcoma sub-type. The initial tumours in these patients included a previous leiomyosarcoma of the uterus, seven other tumours of genito-urinary tract, three breast cancers, two colon cancers and two brain tumours. Additional primary tumours were diagnosed at ages ranging from 31-85 years.

The youngest patient in the study to be diagnosed with a sarcoma and an additional primary cancer (case 1) was known to suffer from neurofibromatosis. One patient had two sarcomas (case 10) and seven patients were diagnosed with carcinoma of the breast in addition to their sarcoma (cases 2, 6, 9, 11, 17, 18 and 29).

A total of 261 patients (125 males and 136 females) and seven cancers were eligible to be included in analysis of risk of developing other malignancies after diagnosis of sarcoma. The observed cancers were in excess of those expected but the excess was not statistically significant (person years at risk = 807, obs = 7, exp = 4.58, RR = 1.5, 95% CI 0.6–3.1, $P = 0.2$). Analysis by sex, age at diagnosis (<60 years and 60 + years) and by histological sub-type of sarcoma similarly did not reveal any significant excesses of second cancers. Exclusion of second malignancies and years of follow-up within 6 months of diagnosis of sarcoma resulted in an excess risk of 1.06 (person years at risk = 692, obs = 4, exp = 3.77, 95% CI 0.3–2.7, $P = 0.5$).

### Discussion

The study reveals that very few younger patients had multiple primary tumours involving sarcomas. Only two patients in the three-year series were under the age of 45 years, and six patients under the age of 60 years at the time of diagnosis of both their malignancies. The youngest case with a double primary cancer was a known case of neurofibromatosis but this syndrome, although registrable under cancer registration schemes, was not recorded on the registration form for any other patient in the study who developed a second primary tumour.

Ascertainment of patients with multiple primary cancers may lead to the identification of families with the Li-Fraumeni syndrome (LFS), a syndrome characterised by the development of sarcomas in children and young adults together with early onset cancers in their close relatives and in which multiple primary tumours frequently occur. Although seven patients in the series had a sarcoma and carcinoma of the breast, a classic combination of cancers in the LFS, only two of these patients were aged under 60 years, the age by which the majority of LFS cancers occur, at diagnosis of both tumours. There were no other obvious combinations of LFS-type cancers, e.g. sarcoma and brain tumour or sarcoma and leukaemia/lymphoma, in any younger patients and it seems unlikely that any other than a small proportion of the study population may have been affected by this syndrome. However, as more than 50 of the study population are still under the age of 60 years, a full family history of cancer for each patient would be necessary to define patients affected by the syndrome and who may be at risk of second malignancy.

It is clear that double primary cancers involving sarcomas are extremely rare in the general population and that few patients with syndromes predisposing to sarcomas and other cancers can be directly identified from cancer registrations. Many double primaries of this nature may simply be chance occurrences in patients of advancing age – data from this same population have shown that incidence of sarcomas in general increases with age to peak between 70–74 years (Hartley *et al.*, 1991).

As a result of the relatively small sample size, short mean period of follow up and high mortality resulting from sarcoma (Hartley *et al.*, 1992), ability to assess risk of developing a further malignancy after diagnosis of sarcoma is limited. However, the establishment of a unique population-based series of sarcomas clearly defined by histopathological review is an important resource for further studies. Peer review is an essential pre-requisite for any investigation of this kind as reclassification of sarcomas is a consistent occurrence (Presant *et al.*, 1986; Harris *et al.*, 1991). Follow-up of the study cohort via the NWRCR and NHSCR over a period of time will enable assessment of risk of subsequent cancer and will also identify those patients with early onset double primaries who may then form the subjects of further work on genetic predisposition to cancer.

We are grateful to the North Western Regional Cancer Registry for the provision of data relating to registration of sarcomas. We would also like to thank the many pathologists who provided material for the study including S. Banik, R.W. Blewitt, W.G. Brown, C.H. Buckley, J. Burns, A.B. Colclough, K.S. Daber, A.S. Day, D.M.H. De Krester, S. Dutt, A.R. Evans, G. Garrett, R. Gillett, J.R. Goepel, I. Gupta, B.N.A. Hamid, D.S. Harry, P.S. Hasleton, C.K. Heffernan, J.R. Helliwell, S.S. Hom-Choudhury, A.C. Hunt, N.N. Jaswon, A.R. Mainwaring, H.B. Marsden, J.A. Morris, H.M. Myat, W.G. Owen, N.L. Reeve, W.H. Richmond, C.M. Starkie, V. Tagore, W.H. Taylor, E.G.F. Tinsley, J.M. Torry, D.M. Vickers, S. Wells, J.S. Whittaker, G. Williams, H.D. Zakhour.

We should like to thank the staffs of the various medical records departments for their help in patient follow up, and the staff of the National Health Service Central Register for flagging and for provision of death notifications.

We are particularly grateful to Ewa Dale who scrutinised the cancer registrations and coordinated the receipt and dispatch of material, and to Joy Hogg who typed the manuscript.

This work was supported by the Cancer Research Campaign.

### References

BIRCH, J.M. (1987). Genetic determinants of cancer in man. In *Biology of Carcinogenesis*, Waring, M.J. & Ponder, B. (eds) pp. 165–189. MTP Press: Lancaster.

BURKE, E., LI, F.P., JANOV, A.J., BATTER, S., GRIER, H. & GOORIN, A. (1991). Cancer in relatives of survivors of childhood sarcoma. *Cancer*, **67**, 1467–1469.

ENZINGER, F.M. & WEISS, S.W. (1988). *Soft Tissue Tumors*. 2nd Edition, C.V. Mosby Co.: St Louis.

EPILOG PLUS (1987). Epicenter Software, Pasadena.

HARRIS, M., HARTLEY, A.L., BLAIR, V., BIRCH, J.M., BANERJEE, S.S., FREEMONT, A.J., MCCLURE, J. & MCWILLIAM, L.J. (1991). Sarcomas in North West England: I Histopathological peer review. *Br. J. Cancer*, **64**, 315–320.

HARTLEY, A.L., BLAIR, V., HARRIS, M., BIRCH, J.M., BANERJEE, S.S., FREEMONT, A.J., MCCLURE, J. & MCWILLIAM, L.J. (1991). Sarcomas in North West England: II Incidence. *Br. J. Cancer*, **64**, 1145–1150.

HARTLEY, A.L., BLAIR, V., HARRIS, M., BIRCH, J.M., BANERJEE, S.S., FREEMONT, A.J., MCCLURE, J. & MCWILLIAM, L.J. (1992). Sarcomas in North West England: III Survival. *Br. J. Cancer*, **66**, 685–691.

HOPE, D.G. & MULVIHILL, J.J. (1981). Malignancy in neurofibromatosis. *Adv. Neurol.*, **29**, 33–56.

LI, F.P., FRAUMENI, J.F., Jr. MULVIHILL, J.J., BLATTNER, W.A., DREYFUS, M.G., TUCKER, M.A. & MILLER, R.W. (1988). A cancer family syndrome in twenty-four kindreds. *Cancer Res.*, **48,** 5358–5362.

MALKIN, D., LI, F.P., STRONG, L.C., FRAUMENI, J.F. Jr., NELSON, C.E., KIM, D.H., KASSEL, J., GRYKA, M.A., BISCHOFF, F.Z., TAINSKY, M.A. & FRIEND, S.H. (1990). Germ line p53 mutations in a familial syndrome of breast cancer, sarcomas, and other neoplasms. *Science*, **250,** 1233–1238.

MALKIN, D., JOLLY, K.W., BARBIER, N., LOOK, A.T., FRIEND, S.H., GEBHARDT, M.C., ANDERSEN, T.I., BØRRESEN, A.-L., LI, F.P., GARBER, J. & STRONG, L.C. (1992). Germline mutations of the p53 tumor-suppressor gene in children and young adults with second malignant neoplasms. *N. Engl. J. Med.*, **326,** 1309–1315.

NWENE, U. & SMITH, A. (1982). Assessing completeness of cancer registration in the North-Western region of England by a method of independent comparison. *Br. J. Cancer*, **46,** 635–639.

PRESANT, C.A., RUSSELL, W.O., ALEXANDER, R.W. & FU, Y.S. (1986). Soft-tissue and bone sarcoma histopathology peer review: The frequency of disagreement in diagnosis and the need for second pathology opinions. The Southeastern Cancer Study Group Experience. *J. Clin. Oncol.*, **4,** 1658–1661.

SANTIBÁÑEZ-KOREF, M.F., BIRCH, J.M., HARTLEY, A.L., MORRIS JONES, P.H., CRAFT, A.W., EDEN, T., CROWTHER, D., KELSEY, A.M. & HARRIS, M. (1991). P53 germline mutations in Li-Fraumeni syndrome. *Lancet*, **338,** 1490–1491.

SCHOTTENFELD, D. (1982). Multiple primary cancers. In *Cancer Epidemiology and Prevention*, Schottenfeld, D. & Fraumeni, J.F. (eds) pp. 1025–1035. W.B. Saunders: Philadelphia.

STRONG, L.C., STINE, M. & NORSTED, T.L. (1987). Cancer in survivors of childhood soft tissue sarcoma and their relatives. *J. Natl Cancer Inst.*, **79,** 1213–1220.

TOGUCHIDA, J., YAMAGUCHI, T., DAYTON, S.H., BEAUCHAMP, R.L., HERRERA, G.E., ISHIZAKI, K., YAMAMURO, T., MEYERS, P.A., LITTLE, J.B., SASAKI, M.S., WEICHSELBAUM, R.R. & YANDELL, D.W. (1992). Prevalence and spectrum of germline mutations of the p53 gene among patients with sarcoma. *N. Engl. J. Med.*, **326,** 1301–1308.

WORLD HEALTH ORGANISATION: ICD-O: International Classification of Diseases for Oncology (1976). World Health Organisation: Geneva.